# Characteristics and clinical course of patients referred to the NST

**DOI:** 10.3389/fnut.2023.1071541

**Published:** 2023-01-26

**Authors:** Jeong Bin Bong, Ji Yeon Chung, So-Yeong Kim, Han Uk Ryu, Byoung-Soo Shin, Hyun Goo Kang

**Affiliations:** ^1^Department of Neurology, Chosun University School of Medicine, Gwangju, Republic of Korea; ^2^Department of Preventive Medicine, College of Medicine, Chosun University, Gwangju, Republic of Korea; ^3^Department of Neurology and Biomedical Research Institute, Jeonbuk National University Medical School and Hospital, Jeonju, Republic of Korea

**Keywords:** nutrition support team, pressure sores grade, malnutrition, albumin level, neurology

## Abstract

**Background and aims:**

The nutrition support team (NST) comprises doctors, nutritionists, pharmacists, and nurses who provide intensive nutritional treatment designed for each patient by evaluating their nutritional status of hospitalized patients. This study aimed to identify the clinical characteristics of patients referred to the NST among those admitted to a tertiary hospital and to understand the factors affecting their clinical course and changes in pressure sore grades.

**Methods:**

This study included 1,171 adult patients aged 18 years or older referred to the NST at a tertiary hospital in a metropolitan city between 1 January 2019 and 31 December 2020. Patients were divided into five age groups, neuro department and non-neuro department, those treated in the intensive care unit (ICU), and those not treated in the ICU. Patients were also compared based on the presence of pressure sores at the time of NST referral and changes in pressure sore grades at the first time of NST referral and discharge (improved pressure sores, no change in pressure sores, and aggravated pressure sores). In addition, this study examined the factors affecting changes in pressure sore grades.

**Results:**

As age increased, the proportion of both low albumin levels and pressure sores significantly increased (*p* < 0.001), and the neuro department showed a significantly lower proportion of low albumin levels and pressure sores (*p* < 0.001). The proportion of patients with pressure sores was higher (64.9%), and this patient group showed significantly higher rates of low albumin levels (*p* < 0.001) and treatment in the ICU (*p* < 0.001). The group with aggravated pressure sore grades had a significantly higher proportion of patients in the surgery department (*p* = 0.009) and those treated in the ICU (*p* < 0.001). Admission to the surgery department was a factor that aggravated the grade of pressure sores [adjusted odds ratio (aOR) = 1.985, 95% confidence interval (CI) = 1.168–3.371]. When patients were not treated in the ICU, the grade of the pressure sores was less likely to worsen (aOR = 0.364, 95% CI = 0.217–0.609).

**Conclusion:**

Pressure sores and low albumin levels are closely related, and the risk of developing and aggravating pressure sores is particularly high in patients in the surgery department and those receiving ICU treatment. Therefore, it is necessary to actively implement NST referral to ensure that overall nutrition, including albumin, is well supplied, especially for patients in the surgery department and treated in the ICU, as they are at high risk of pressure sore development and aggravation. Moreover, since low albumin levels frequently occur in elderly patients, it is necessary to consider including the elderly in the indications for referral to the NST.

## 1. Introduction

Malnutrition is a common problem among hospitalized patients. Previous studies have reported that 40% of all hospitalized patients had risk factors associated with malnutrition, and 75% became more malnourished during hospitalization (1). Malnutrition is a particularly critical problem in patients with severe diseases and long–term inpatients. It is associated with nutrition-related complications and a poor prognosis during hospitalization and after discharge. In addition, previous studies have reported a relationship between serum albumin and pressure sores, and proper nutritional support is essential to prevent pressure sores (2).

The nutrition support team (NST) aims to implement intensive nutritional treatment for each patient by pre–assessing the malnutrition status and the possibility of malnutrition by evaluating the nutritional status of hospitalized patients. It is an expert group comprising doctors, nutritionists, pharmacists, and nurses. Developed countries, such as the United States, began organizing the NST in the late 1960s to provide appropriate nutritional support for patients needing parenteral and enteral nutrition (PN and EN). South Korea started the NST with the voluntary participation of experts active in nutrition support in the late 1990s. As the “intensive nutrition treatment fee” became an item of health insurance in August 2014, the importance of nutritional treatment was further highlighted. Since then, more hospitals have installed and operated NST, and awareness regarding the importance of nutrition management for inpatients has been increasing. However, studies on the characteristics and treatment effects of patients referred to the NST are insufficient.

Therefore, this study aimed to investigate the direction and focus of NST activities by identifying the clinical characteristics of patients referred to the NST among those hospitalized in tertiary hospitals and to understand the factors influencing their clinical course and changes in the grade of pressure sores.

## 2. Materials and methods

### 2.1. Study participants

The participants were 1,490 adult patients (18 years or older) referred to the NST at least twice, with an interval of 1 week at tertiary hospitals in the metropolitan city between 1 January 2019 and 31 December 2020. After excluding 309 patients who received more than 75% of the required caloric supply at the time of referral and 10 patients with insufficient data for nutritional assessment, the data of 1,171 patients were used. Information on the referred patients was retrospectively analyzed using electronic medical records and NST patient management sheets. This study was approved by the hospital’s Institutional Review Board (2022-09-001). The requirement for consent was waived due to the study’s retrospective nature (2022-09-001).

### 2.2. Contents and methods

This study included 1,171 patients referred to the NST of a tertiary hospital at least twice with an interval of 1 week. The targets of NST referral were those who satisfied at least one of the following criteria: (1) serum albumin ≤ 3.0 g/dl, (2) receiving EN; (3) receiving PN; (4) receiving treatment in an intensive care unit (ICU); and (5) when it was determined that intensive nutritional treatment was necessary based on the medical opinion of the physician in charge.

This study analyzed the patients’ clinical characteristics and course according to sex, age, presence of pressure sores, changes in the grade of pressure sores at the first time of NST referral and discharge, treatment in the ICU, and clinical department. Clinical characteristics included sex, age, height, weight, body mass index (BMI), clinical department, whether the physician in charge was a member of an NST (limited to those who had completed training recognized by the Health Insurance Review and Assessment Service), whether EN or PN was supplied, whether the patient could eat independently, fasting or not, the presence of pressure sores, the grade of pressure sores at the first time of NST referral, laboratory findings at the time of NST referral (including albumin and cholesterol), low albumin levels (serum albumin ≤ 3.0 g/dl), and difficulty chewing and swallowing. The clinical course included the number of weeks until reaching the target calorie when 75% or more of the required calorie was supplied, based on body weight, whether the grade of pressure sores aggravated at discharge, and whether to be treated in the ICU. If a patient was treated in the ICU, the Acute Physiology and Chronic Health Evaluation II (APACHE II) score was used.

Age was divided into five groups: 18–50, 50–59, 60–69, 70–79, and ≥ 80 years. Pressure sores were determined by referring to the pressure sore evaluation sheet in the medical record. The pressure sores were classified into five grades (0–4). The grades at the first NST referral and that at discharge were compared, and classified into three groups: aggravation, improvement, and no change, the differences between the three groups were compared. The departments were compared by classifying them into a neuro department (neurology and neurosurgery) and a non-neuro department (allergy and clinical immunology, cardiology, endocrinology, gastroenterology, hemato–oncology, infectious disease, nephrology, rheumatology, pulmonology, chest surgery, general surgery, orthopedic surgery, and plastic surgery). They were also classified into internal medicine departments (allergy and clinical immunology, cardiology, endocrinology, gastroenterology, hemato–oncology, infectious disease, nephrology, rheumatology, pulmonology, and neurology) and surgery departments (chest surgery, general surgery, orthopedic surgery, plastic surgery, and neurosurgery).

### 2.3. Data analysis methods

First, the demographics, nutritional status, general conditions, and laboratory findings were compared between females and males, neuro and non-neuro departments, those with and without pressure sore, and those with and without ICU treatment. Second, age was divided into five categories, and demographics, nutritional status, general condition, and laboratory findings were compared according to age groups. Third, changes in pressure sore grade during admission were divided into three categories, and demographics, nutritional status, general conditions, and laboratory findings were compared according to these categories. Pearson’s chi-square or Fisher’s exact test was used for categorical variables, and the *t*-test or One-way ANOVA was used for continuous variables. Pearson’s chi-square test was performed, and when the expected frequency of each cell was less than 5, more than 20% of the cells were interpreted as Fisher’s exact test values. In addition, multinomial logistic regression analysis was used to examine the factors that improved or aggravated the sore stage changes. Statistical significance was set at *P* < 0.05 (two-tailed). All statistical analyses were performed using SPSS (version 26.0; IBM Corp., Armonk, NY, USA).

## 3. Results

This study divided 1,171 patients referred to the NST into five age groups (18–50, 50–59, 60–69, 70–79, and ≥ 80 years old or older) and analyzed them to understand the differences according to age (Table 1). The results showed that BMI decreased significantly with an increase in age (*p* < 0.001). In the younger age groups, more NST referrals were made by the surgery department than by the internal medicine department. In the older age groups, more NST referrals were made by the internal medicine department than by the surgery department. The rate of low albumin levels and the presence of pressure sores significantly increased with age (*p* < 0.001). The APACHE II score at the time of ICU admission also significantly increased (*p* = 0.007) with increasing age. In laboratory tests, serum albumin and total cholesterol levels decreased significantly (*p* < 0.001) with age.

**TABLE 1:** Differences of patient characteristics according to age groups.

	<50 (*n* = 123)	50–59 (*n* = 142)	60–69 (*n* = 222)	70–79 (*n* = 352)	≥ 80 (*n* = 332)	*P-value*
Age (mean)	40.1 ± 8.92	55.5 ± 2.69	64.7 ± 2.90	74.9 ± 2.86	84.3 ± 3.76	
Sex (male)	84 (68.3)	108 (76.1)	142 (64.0)	201 (57.1)	181 (54.5)	<0.001
Height	168.47 ± 9.33	166.67 ± 7.53	165.14 ± 7.96	162.99 ± 8.47	161.58 ± 9.25	<0.001
Weight	66.96 ± 18.88	64.31 ± 11.21	61.79 ± 11.23	59.19 ± 10.54	56.72 ± 11.82	<0.001
BMI	23.31 ± 5.21	23.16 ± 3.89	22.59 ± 3.46	22.27 ± 3.52	21.60 ± 3.50	<0.001
Department						
Surgery	74 (60.2)	86 (60.6)	123 (55.4)	147 (41.8)	120 (36.1)	<0.001
Internal medicine	49 (39.8)	56 (39.4)	99 (44.6)	205 (58.2)	212 (63.9)	
Non-neuro*	55 (44.7)	56 (39.4)	111 (50.0)	196 (55.7)	207 (62.3)	<0.001
Neuro*	68 (55.3)	86 (60.6)	111 (50.0)	156 (44.3)	125 (37.7)	
Low albumin**	31 (25.2)	42 (29.6)	82 (36.9)	138 (39.2)	155 (46.7)	<0.001
EN	46 (37.4)	49 (34.5)	82 (36.9)	125 (35.5)	124 (37.3)	0.968
PN	75 (61.0)	81 (57.0)	117 (52.7)	185 (52.6)	187 (56.3)	0.467
Spontaneous feeding	16 (13.0)	21 (14.8)	31 (14.0)	51 (14.5)	51 (14.5)	0.975
NPO	67 (54.5)	74 (52.1)	108 (48.6)	186 (52.8)	165 (49.7)	0.767
Ascite	2 (1.6)	2 (1.4)	0 (0.0)	1 (0.3)	1 (0.3)	0.135
Edema	2 (1.6)	8 (5.6)	9 (4.1)	19 (5.4)	18 (5.4)	0.439
Jaundice	2 (1.6)	2 (1.4)	1 (0.5)	1 (0.3)	1 (0.3)	0.296
Dialysis	5 (4.1)	9 (6.3)	11 (5.0)	14 (4.0)	9 (2.7)	0.424
Bad appetite	12 (9.8)	17 (12.0)	25 (11.3)	37 (10.5)	45 (13.6)	0.719
Difficulty chewing	43 (35.0)	44 (31.0)	80 (36.0)	117 (33.2)	115 (34.6)	0.881
Difficulty swallowing	32 (26.0)	40 (28.2)	65 (29.3)	92 (26.1)	88 (26.5)	0.923
Diarrhea	4 (3.3)	1 (0.7)	9 (4.1)	3 (0.9)	4 (1.2)	0.023
Constipation	1 (0.8)	1 (0.7)	1 (0.5)	0 (0.0)	1 (0.3)	0.618
Pressure sore	57 (46.3)	83 (58.5)	129 (58.1)	241 (68.5)	250 (75.3)	<0.001
Pressure sore grade at NST referral						
Grade 0	5 (8.6)	5 (5.8)	7 (5.1)	8 (3.2)	9 (3.5)	
Grade 1	41 (70.7)	57 (66.3)	95 (68.8)	175 (69.2)	166 (64.6)	
Grade 2	9 (15.5)	19 (22.1)	35 (25.4)	60 (23.7)	76 (29.6)	0.322
Grade 3	3 (5.2)	4 (4.7)	1 (0.7)	7 (2.8)	4 (1.6)	
Grade 4	0 (0.0)	1 (1.2)	0 (0.0)	3 (1.2)	2 (0.8)	
Changes in pressure sore grades***						
Improved	2 (3.4)	4 (4.7)	2 (1.4)	7 (2.8)	10 (3.9)	
No change	47 (81.0)	77 (89.5)	123 (89.1)	227 (89.7)	223 (86.8)	0.494
Aggravated	9 (15.5)	5 (5.8)	13 (9.4)	19 (7.5)	24 (9.3)	
ICU treatment	29 (23.6)	36 (25.4)	62 (27.9)	99 (28.1)	100 (30.1)	0.658
APACHE II score	9.59 ± 9.48	12.82 ± 7.28	14.51 ± 8.24	15.51 ± 7.10	15.06 ± 7.29	0.007
Length of ICU stay	28.03 ± 25.35	18.53 ± 11.42	27.06 ± 29.38	25.88 ± 24.43	21.70 ± 16.06	0.199
Average number of weeks to reach the target calories	1.63 ± 0.94	1.70 ± 0.99	1.58 ± 0.82	1.76 ± 2.54	1.47 ± 1.05	0.684
Physicians received NST training	37 (30.1)	44 (31.0)	47 (21.2)	82 (23.3)	113 (34.0)	0.003
Laboratory findings						
WBC (10^3^/μL)	11.43 ± 5.78	10.79 ± 4.71	11.15 ± 5.01	11.10 ± 4.76	11.27 ± 6.21	0.882
Hb (g/dL)	10.82 ± 2.47	10.65 ± 2.21	10.67 ± 2.26	10.59 ± 2.12	10.45 ± 2.00	0.536
Na (mEq/L)	142.00 ± 7.31	139.33 ± 6.43	141.15 ± 7.09	139.51 ± 6.69	138.99 ± 5.85	<0.001
K (mEq/L)	3.71 ± 0.65	3.78 ± 0.57	3.67 ± 0.62	3.72 ± 0.62	3.67 ± 0.64	0.389
Cl (mEq/L)	106.18 ± 7.86	103.95 ± 6.34	105.35 ± 7.25	104.45 ± 7.15	104.24 ± 6.41	0.026
Ca (mEq/L)	7.99 ± 0.94	8.22 ± 0.62	8.13 ± 0.73	8.06 ± 0.73	7.91 ± 0.69	0.024
Mg (mEq/L)	2.03 ± 0.36	2.16 ± 0.35	2.07 ± 0.36	2.12 ± 0.33	2.14 ± 0.37	0.197
P (mg/dL)	2.66 ± 1.19	3.47 ± 1.28	2.89 ± 1.06	3.02 ± 1.50	2.96 ± 1.25	0.108
ALT (U/L)	42.57 ± 66.65	55.89 ± 159.17	39.66 ± 96.64	37.40 ± 97.13	31.08 ± 63.02	0.143
AST (U/L)	68.63 ± 145.59	83.50 ± 270.14	62.02 ± 157.15	61.34 ± 238.28	44.33 ± 52.62	0.280
Glucose (mg/dL)	136.95 ± 70.90	141.86 ± 64.07	145.64 ± 75.26	142.27 ± 71.21	128.07 ± 61.15	0.021
Albumin (g/dL)	3.34 ± 0.62	3.34 ± 0.59	3.22 ± 0.59	3.18 ± 0.50	3.08 ± 0.50	<0.001
Cholesterol (mg/dL)	155.57 ± 53.43	151.63 ± 49.47	138.01 ± 46.25	133.65 ± 41.27	129.85 ± 41.45	<0.001
Triglyceride (mg/dL)	148.92 ± 109.84	124.70 ± 79.74	129.52 ± 99.45	109.59 ± 81.05	89.51 ± 44.95	<0.001
CRP (mg/dL)	8.38 ± 8.18	8.05 ± 8.89	9.45 ± 9.05	9.12 ± 8.55	9.24 ± 8.50	0.532
BUN (mg/dL)	19.22 ± 12.65	20.74 ± 14.51	21.64 ± 14.38	26.50 ± 17.95	25.85 ± 17.21	<0.001
Cr (mg/dL)	0.97 ± 1.10	1.07 ± 1.44	1.01 ± 1.22	1.09 ± 1.08	1.03 ± 0.97	0.854

Values are the number of patients (%) or mean ± standard deviation unless otherwise indicated. *Neuro = Neurology and Neurosurgery, Non-neuro = other departments, **Low albumin = serum albumin ≤ 3.0 g/dL, ***Changes in pressure sore grades at the first time of NST referral and discharge. BMI, body mass index; EN, enteral nutrition; PN, parenteral nutrition; NPO, nil per os or nothing by mouth; ICU, intensive care unit; APACHE II, acute physiology and chronic health evaluation II; NST, nutrition support team; WBC, white blood cell; Hb, hemoglobin; CRP, C-reactive protein; BUN, blood urea nitrogen; Cr, creatinine.

This study divided patients into the neuro and non-neuro departments and compared them to examine the differences according to department (Table 2). The neuro department accounted for 46.6% of patients, and this group was younger (67.4 ± 14.6 vs. 71.6 ± 14.0) and had a higher BMI (23.30 ± 3.85 vs. 21.52 ± 3.57). The neuro department had fewer cases of low albumin levels (14.1 vs. 59.4%), whereas the rate of EN was higher (50.4 vs. 24.2%). patients had a lower proportion of PN (42.9 vs. 65.8%), spontaneous feeding (6.2 vs. 21.8%), and fasting (46.3 vs. 55.5%). While the neuro department had a lower proportion of ascites (0 vs. 1.0%), edema (2.0 vs. 7.2%), jaundice (0 vs. 1.1%), dialysis (2.7 vs. 5.3%), and poor appetite (3.7 vs. 18.6%), it had a higher proportion of difficulty chewing (48.4 vs. 21.6%) and swallowing (41.2 vs. 14.7%). Patients in the neuro department had a lower rate of pressure sores (59.7 vs. 69.4%). When patients were admitted to the ICU, the APACHE II score of the neuro department patients was higher than that of the non-neuro department patients (15.66 ± 8.17 vs. 13.20 ± 7.28). However, the length of stay in the ICU did not differ between neuro and non-neuro departments. The neuro department patients took more weeks to reach the target calories than the non-neuro department patients (mean number of weeks = 2.10 ± 2.59 vs. 1.41 ± 0.87). Moreover, the neuro department had significantly fewer cases in which the supplied calories 1 week after the NST referral were higher than those at the time of the first NST referral (67.0 vs. 79.4%). In addition, neuro department patients had significantly lower amounts of improved calories (12.033 ± 26.1 vs. 27.861 ± 36.12) (*p* < 0.001). Fewer physicians in the neuro department received NST training (17.2 vs. 36.6%). Plasma hemoglobin (Hb), serum sodium (Na), albumin, and total cholesterol levels of patients in the non-neuro department were significantly lower than those of patients in the neuro department group (*p* < 0.001).

**TABLE 2:** Differences of patient characteristics according to departments.

	Neuro* (*n* = 546)	Non-neuro* (*n* = 625)	*P-value*
Age (mean)	67.4 ± 14.58	71.6 ± 13.96	<0.001
Sex (male)	321 (58.8)	395 (63.2)	0.123
Height	163.94 ± 11.10	162.75 ± 16.13	0.148
Weight	63.04 ± 13.46	57.61 ± 12.56	<0.001
BMI	23.30 ± 3.85	21.52 ± 3.57	<0.001
Low albumin**	77 (14.1)	371 (59.4)	<0.001
EN	275 (50.4)	151 (24.2)	<0.001
PN	234 (42.9)	411 (65.8)	<0.001
Spontaneous feeding	34 (6.2)	136 (21.8)	<0.001
NPO	253 (46.3)	347 (55.5)	0.002
Ascite	0 (0.0)	6 (1.0)	0.033
Edema	11 (2.0)	45 (7.2)	<0.001
Jaundice	0 (0.0)	7 (1.1)	0.017
Dialysis	15 (2.7)	33 (5.3)	0.029
Bad appetite	20 (3.7)	116 (18.6)	<0.001
Difficulty chewing	264 (48.4)	135 (21.6)	<0.001
Difficulty swallowing	225 (41.2)	92 (14.7)	<0.001
Diarrhea	7 (1.3)	14 (2.2)	0.218
Constipation	2 (0.4)	2 (0.3)	1.000
Pressure sore	326 (59.7)	434 (69.4)	<0.001
**Pressure sore grade at NST referral**			
Grade 0	19 (5.6)	15 (3.3)	
Grade 1	237 (69.3)	297 (66.0)	
Grade 2	77 (22.5)	122 (27.1)	0.316
Grade 3	7 (2.0)	12 (2.7)	
Grade 4	2 (0.6)	4 (0.9)	
Changes in pressure sore grades***			
Improved	9 (2.6)	16 (3.6)	
No change	301 (88.0)	396 (88.0)	0.702
Aggravated	32 (9.4)	33 (8.4)	
ICU treatment	138 (25.3)	188 (30.1)	0.067
APACHE II score	15.66 ± 8.17	13.20 ± 7.28	0.008
Length of ICU stay	22.22 ± 21.88	25.66 ± 22.63	0.170
Average number of weeks to reach the target calories	2.10 ± 2.59	1.41 ± 0.87	0.003
Number of patients who improved their calories	366 (67.0)	496 (79.4)	<0.001
Amounts of improved calories	12.033 ± 26.1	27.861 ± 36.12	<0.001
Physicians received NST training	94 (17.2)	229 (36.6)	<0.001
**Laboratory findings**			
WBC (10^3^/μL)	11.12 ± 4.36	11.18 ± 6.09	0.846
Hb (g/dL)	11.22 ± 2.19	10.05 ± 1.99	<0.001
Na (mEq/L)	141.38 ± 7.16	138.63 ± 5.88	<0.001
K (mEq/L)	3.72 ± 0.58	3.69 ± 0.65	0.361
Cl (mEq/L)	106.12 ± 6.96	103.42 ± 6.74	<0.001
Ca (mEq/L)	8.31 ± 0.53	7.92 ± 0.77	<0.001
Mg (mEq/L)	2.06 ± 0.25	2.14 ± 0.38	0.003
P (mg/dL)	3.72 ± 2.40	2.95 ± 1.23	0.006
ALT (U/L)	26.44 ± 30.30	49.67 ± 127.99	<0.001
AST (U/L)	40.04 ± 39.92	77.58 ± 246.92	<0.001
Glucose (mg/dL)	143.70 ± 65.43	133.53 ± 71.06	0.110
Albumin (g/dL)	3.49 ± 0.49	2.94 ± 0.46	<0.001
Cholesterol (mg/dL)	157.33 ± 42.62	118.05 ± 38.76	<0.001
Triglyceride (mg/dL)	112.55 ± 84.94	113.17 ± 78.49	0.915
CRP (mg/dL)	6.59 ± 7.19	11.11 ± 9.22	<0.001
BUN (mg/dL)	21.74 ± 14.82	25.84 ± 17.47	<0.001
Cr (mg/dL)	0.97 ± 1.130	1.10 ± 1.12	0.041

Values are the number of patients (%) or mean ± standard deviation unless otherwise indicated. *Neuro = Neurology and Neurosurgery, Non-neuro = other departments, **Low albumin = serum albumin ≤ 3.0 g/dL, ***Changes in pressure sore grades at the first time of NST referral and discharge. BMI, body mass index; EN, enteral nutrition; PN, parenteral nutrition; NPO, nil per os or nothing by mouth; ICU, intensive care unit; APACHE II, acute physiology and chronic health evaluation II; NST, nutrition support team; WBC, white blood cell; Hb, hemoglobin; CRP, C-reactive protein; BUN, blood urea nitrogen; Cr, creatinine.

This study divided the patients into a group with pressure sores and a group without pressure sores and compared both groups to examine the differences according to the presence of pressure sores (Table 3). Among the patients referred to the NST, 64.9% had pressure sores. Those with pressure sores were older (71.7 ± 13.4 vs. 65.8 ± 15.4) and had a lower BMI (22.07 ± 3.85 vs. 22.89 ± 3.67). Furthermore, the proportion of patients with pressure sores was higher in the internal medicine department than in the surgery department (60.3 vs. 39.7%, *p* < 0.001). The neuro department had a lower proportion of patients with pressure sores than the non-neuro department (42.9 vs. 57.1%, *p* < 0.001). Patients with pressure sores had higher rates of low albumin levels (43.8 vs. 28.0%) and EN (39.2 vs. 31.1%) and a lower rate of spontaneous feeding (12.0 vs. 19.2%). Additionally, the rates of accompanying edema (6.2 vs. 2.2%), difficulty chewing (37.0 vs. 28.7%), and swallowing (29.1 vs. 23.4%) were higher in patients with pressure sores. In contrast, the rate of poor appetite was lower in patients with pressure sores (9.5 vs. 15.6%). Patients with pressure sores (89.7%) had a higher rate of no change in the grade of pressure sores at the first NST referral and at the time of discharge. In contrast, patients without pressure sores were more likely to have aggravated pressure sores at discharge (50%, *p* < 0.001). A higher proportion of patients with pressure sores were treated in the ICU (31.3 vs. 21.4%) for a longer (27.24 ± 23.96 vs. 16.00 ± 14.42). The group with pressure sores had lower plasma Hb (10.48 ± 2.12 vs. 10.81 ± 2.24), serum albumin (3.13 ± 0.52 vs. 3.32 ± 0.57), and total cholesterol levels (133.33 ± 46.55 vs. 145.07 ± 41.54) than that without pressure sores.

**TABLE 3:** Differences of patient characteristics according to pressure sores.

	With pressure sore (*n* = 760)	Without pressure sore (*n* = 411)	*P-value*
Age (mean)	71.7 ± 13.39	65.8 ± 15.40	<0.001
Sex (male)	460 (60.5)	256 (62.3)	0.555
Height	163.32 ± 13.72	163.28 ± 14.53	0.962
Weight	59.25 ± 13.29	61.81 ± 13.08	0.002
BMI	22.07 ± 3.85	22.89 ± 3.67	<0.001
**Department**			
Surgery	302 (39.7)	248 (60.3)	<0.001
Internal medicine	458 (60.3)	163 (39.7)	
Non-neuro*	434 (57.1)	191 (46.5)	<0.001
Neuro*	326 (42.9)	220 (53.5)	
Low albumin**	333 (43.8)	115 (28.0)	<0.001
EN	298 (39.2)	128 (31.1)	0.006
PN	431 (56.7)	214 (52.1)	0.127
Spontaneous feeding	91 (12.0)	79 (19.2)	0.001
NPO	389 (51.2)	211 (51.3)	0.960
Ascite	3 (0.4)	3 (0.7)	0.429
Edema	47 (6.2)	9 (2.2)	0.002
Jaundice	2 (0.3)	5 (1.2)	0.056
Dialysis	29 (3.8)	19 (4.6)	0.506
Bad appetite	72 (9.5)	64 (15.6)	0.002
Difficulty chewing	281 (37.0)	118 (28.7)	0.004
Difficulty swallowing	221 (29.1)	96 (23.4)	0.035
Diarrhea	13 (1.7)	8 (1.9)	0.772
Constipation	2 (0.3)	2 (0.5)	0.616
Changes in pressure sore grades***			
Improved	24 (3.2)	1 (3.1)	
No change	682 (89.7)	15 (46.9)	<0.001
Aggravated	54 (7.1)	16 (50.0)	
ICU treatment	238 (31.3)	88 (21.4)	<0.001
APACHE II score	14.16 ± 8.02	14.89 ± 7.10	0.497
Length of ICU stay	27.24 ± 23.96	16.00 ± 14.42	<0.001
Average number of weeks to reach the target calories	1.64 ± 1.80	1.60 ± 1.195	0.817
Number of patients who improved their calories	565 (74.3)	297 (72.3)	0.441
Amounts of improved calories	20.27 ± 32.43	20.86 ± 33.53	0.769
Physicians received NST training	212 (27.9)	111 (27.0)	0.746
**Laboratory findings**			
WBC (10^3/μL)	11.29 ± 5.66	10.89 ± 4.71	0.223
Hb (g/dL)	10.48 ± 2.12	10.81 ± 2.24	0.015
Na (mEq/L)	139.74 ± 6.75	140.23 ± 6.45	0.237
K (mEq/L)	3.67 ± 0.60	3.77 ± 0.65	0.007
Cl (mEq/L)	104.44 ± 6.99	105.14 ± 6.93	0.101
Ca (mEq/L)	7.99 ± 0.69	8.14 ± 0.83	0.028
Mg (mEq/L)	2.14 ± 0.37	2.05 ± 0.30	0.004
P (mg/dL)	2.97 ± 1.37	3.06 ± 1.12	0.560
ALT (U/L)	39.36 ± 96.77	39.69 ± 95.83	0.822
AST (U/L)	55.07 ± 149.96	69.37 ± 232.82	0.261
Glucose (mg/dL)	137.44 ± 69.09	139.80 ± 67.89	0.574
Albumin (g/dL)	3.13 ± 0.52	3.32 ± 0.57	<0.001
Cholesterol (mg/dL)	133.33 ± 46.55	145.07 ± 41.54	<0.001
Triglyceride (mg/dL)	111.61 ± 76.15	115.18 ± 91.16	0.560
CRP (mg/dL)	9.59 ± 8.76	7.95 ± 8.31	0.002
BUN (mg/dL)	24.92 ± 16.96	22.11 ± 15.20	0.005
Cr (mg/dL)	1.02 ± 1.08	1.08 ± 1.21	0.324

Values are the number of patients (%) or mean ± standard deviation unless otherwise indicated. *Neuro = Neurology and Neurosurgery, Non-neuro = other departments, **Low albumin = serum albumin ≤ 3.0 g/dL, ***Changes in pressure sore grades at the first time of NST referral and discharge. BMI, body mass index; EN, enteral nutrition; PN = parenteral nutrition; NPO, nil per os or nothing by mouth; ICU, intensive care unit; APACHE II, acute physiology and chronic health evaluation II; NST, nutrition support team; WBC, white blood cell; Hb, hemoglobin; CRP, C-reactive protein; BUN, blood urea nitrogen; Cr, creatinine.

This study divided 792 patients whose pressure sore grades were confirmed using their medical records among all patients into cases of improved pressure sore grades, cases without a change in pressure sore grades, and cases of aggravated pressure sores grades to examine the difference between target patients’ according to the change in the pressure sore grades at the first time of NST referral and discharge (Supplementary Table 1). The group with aggravated pressure sore grades had significantly higher body weight and BMI than the other groups (*p* = 0.003 and 0.002, respectively), and the proportion of patients in the surgery department was also higher (*p* = 0.009). In addition, the presence of ascites was significantly higher in the group with aggravated pressure sore grades than in the other groups (*p* = 0.002), and the rate of treatment in the ICU was significantly higher in this group than in the other groups (*p* < 0.001). Multinomial logistic regression analysis was performed using significant variables (*p* < 0.05) in the crossover analysis and one-way analysis of variance to examine the factors affecting the change in the pressure sore grades (Table 4). The results showed that patients in the surgery department had a higher risk of aggravating pressure sores [adjusted odds ratio (aOR) = 1.985, 95% confidence interval (CI) = 1.168–3.371]. In contrast, patients not treated in the ICU had a lower risk of aggravating pressure sores (aOR = 0.364, 95% CI = 0.217–0.609). Moreover, the results of the univariate analysis revealed that not being treated in the ICU significantly improved the pressure sore grades (crude OR = 3.561, 95% CI = 1.056–12.035); however, this variable was not significant when adjusted for other variables.

**TABLE 4:** Factors influencing changes in the pressure sore grades.

	Sore improvement				Sore aggravation			
	Crude OR (95% CI)	*P-value*	Adjusted OR^†^ (95% CI)	*p-value*	Crude OR (95% CI)	*P-value*	Adjusted OR^†^ (95% CI)	*p-value*
Age	1.015 (0.983–1.049)	0.392	1.023 (0.987–1.061)	0.213	0.994 (0.976–1.012)	0.514	1.006 (0.986–1.027)	0.538
Male	0.698 (0.314–1.551)	0.377	0.663 (0.186–2.150)	0.463	1.234 (0.736–2.068)	0.425	1.164 (0.535–2.535)	0.701
Weight	1.001 (0.980–1.043)	0.505	1.010 (0.922–1.107)	0.832	1.031 (1.013–1.050)	0.001	1.008 (0.951–1.068)	0.788
BMI	1.058 (0.958–1.169)	0.266	1.045 (0.796–1.373)	0.749	1.107 (1.043–1.176)	0.001	1.072 (0.897–1.282)	0.443
Glucose	0.995 (0.987–1.003)	0.195	0.994 (0.986–1.002)	0.172	1.003 (1.000–1.006)	0.042	1.002 (0.999–1.005)	0.217
Surgery department	1.460 (0.656–3.247)	0.354	1.167 (0.683–3.829)	0.275	2.109 (1.282–3.467)	0.003	1.985 (1.168–3.371)	0.011
Without ICU treatment	3.565 (1.056–12.035)	0.041	3.319 (0.974–11.309)	0.055	0.386 (0.235–0.635)	<0.001	0.364 (0.217–0.609)	<0.001

Values are odds ratios (95% confidence intervals). BMI, body mass index; ICU, intensive care unit.

^†^OR for changes in pressure sore grades: OR adjusted for age, sex, weight, BMI, glucose, surgery department, and absence of ICU treatment.

When classifying the characteristics according to sex (Supplementary Table 2), there were more males than females (61.1 vs. 38.9%), and the male patients were younger (68.2 ± 14.4 vs. 71.8 ± 14.2). More male patients were referred to the NST in the surgery department (49.3 vs. 43.3%), whereas more female patients were referred to the NST in the internal medicine department (50.7 vs. 56.7%). Females had more edema (3.4 vs. 7.0%) and difficulty swallowing (24.6 vs. 31.0%) than males. The Hb level of females was lower (10.85 ± 2.22 vs. 10.21 ± 2.01), and their serum total cholesterol level was higher (134.00 ± 43.74 vs. 142.63 ± 46.88).

The target patients were divided into those who were treated in the ICU (ICU group) and those who were not treated in the ICU (no ICU group), and both groups were analyzed to evaluate the differences according to ICU treatment (Supplementary Table 3). The results showed that the ICU group had a higher proportion of low albumin levels (45.4 vs. 35.5%), PN (68.7 vs. 49.8%), and fasting (65.0 vs. 45.9%), and a lower proportion of EN (28.5 vs. 39.4%) and spontaneous feeding (7.7 vs. 17.2%). The ICU group also had a higher proportion of patients with edema (8.0 vs. 3.6%) and dialysis (6.7 vs. 3.1%). In contrast, the ICU group had a lower proportion of patients with poor appetite (6.4 vs. 13.6%) and difficulty chewing (26.7 vs. 36.9%). In addition, the ICU group had a higher proportion of patients with pressure sores (73.0 vs. 61.8%) and aggravated pressure sore grades (14.4 vs. 5.9%). In contrast, the possibility of improving the pressure sore grade was lower (1.1 vs. 4.2%). Furthermore, the ICU group showed significantly higher white blood counts (12.07 ± 6.22 vs. 10.80 ± 4.93) and a significantly lower Hb level (10.33 ± 2.18 vs. 10.70 ± 2.15). The ICU group also had significantly higher serum albumin and C-reactive protein levels (*p* < 0.001).

## 4. Discussion

This study describes that low albumin levels frequently occur in elderly patients and that pressure sores and low albumin levels are closely related. We found that the risk of aggravating pressure sores was particularly high in patients in the surgery department and those receiving ICU treatment.

This study found that the proportion of patients with low albumin levels increases with age. As previous studies have shown that healthy older people have normal albumin levels, age in itself is not a pathophysiological mechanism for hypoalbuminemia (3). In contrast, patients can develop hypoalbuminemia due to a combination of inflammatory responses and a lack nutritional intake (4). Therefore, the results of this study were different from those of previous studies, which showed that age was not associated with hypoalbuminemia, possibly because this study targeted patients rather than healthy individuals. This study also showed that the proportion of accompanying pressure sores increases with age. Mathus–Vliegen reported that a decrease in albumin levels in elderly patients suggested malnutrition, and the presence of accompanying pressure sores could lead to a very poor prognosis (5). Therefore, to prevent a poor prognosis, it is necessary to pay more attention to preventing low albumin levels in elderly patients. To achieve this goal, it is also necessary to actively implement NST referrals. South Korea began to apply the treatment cost of “Therapy by nutrition support team” in August 2014 through the announcement of the Ministry of Health and Welfare (6), and the indication criteria for selecting suitable participants for NST treatment were determined. While the serum albumin concentration was an indication, old age was not included. The elderly group may be included in the NST treatment group according to the judgment of the physician in charge because the results of this study confirm that old age can be an indication for NST treatment.

Among patients admitted to the neuro departments of a tertiary hospital, the proportion of acute diseases such as cerebral infarction, cerebral hemorrhage, and encephalitis is higher than that of chronic and intractable neurological disorders (7). For this reason, the proportion of low albumin levels was believed to be low in the neuro department group. Moreover, the proportion of EN was high because of the high rate of swallowing difficulties due to decreased consciousness and brain lesions. In contrast, the proportion of PN was low in the neuro department group, because hemodynamic instability or digestive problems were less common than those in the internal medicine department patients. At the time of admission to the ICU, the APACHE II score of the neuro department patients was higher. Among the 12 acute physiology items used to calculate the APACHE II score, the Glasgow Coma Scale (GCS) for evaluating neurological status ranges from 0 to 12 as a single item (8). The APACHE II score of the neuro department patients was believed to be high because GCS accounted for a relatively large proportion. When comparing the first and second NST referrals, there were fewer cases of improved caloric intake and caloric values in the neuro department. More weeks were required to supply more than 75% of the required amount. Additional studies are needed to understand whether this is associated with the characteristics of the neuro department and/or other external factors, such as the proportion of NST–trained physicians in charge.

The Waterlow tool is used to assess the risk of pressure sores. This tool consists of 11 elements: BMI, visual risk area/skin condition, sex, age, nutritional status, urine and feces control, mobility, and unique risk factors, including tissue malnutrition, neurological deficit, major surgery or trauma, and drugs (9). In this study, the group with pressure sores had a higher proportion of elderly individuals, low BMI, low albumin level, and EN; these four items were consistent with the Waterlow score. Furthermore, in the Waterlow score, the risk of developing pressure sores increased when there was a neurological defect, major surgery, or trauma as unique risk factors. However, in this study, in the neuro and surgery departments, the proportion of patients without pressure sores at the time of the first referral was higher than that of patients with pressure sores at the time of the first referral. However, the group with aggravated pressure sore grades had a significantly higher proportion of patients in the surgery department. The results of the multivariate analysis regarding the factors influencing the aggravation of pressure sore grade also confirmed that patients in the surgery departments were an exacerbation factor. Since the surgery department had a higher proportion of acute diseases and younger patients, as confirmed by the analysis of the effects of age (Table 1), they were more likely not to have pressure sores at the time of first referral, that is, at the beginning of the admission. However, patients in the surgery department consumed more calories because they were more likely to experience trauma or have undergone surgery. They are also more likely to develop new pressure sores or worsen existing ones because of the difficulty in changing positions due to multiple traumas. According to the Waterlow score evaluation factor, this is consistent with the increased risk of pressure sores in cases of major surgery or trauma. Therefore, it is possible to prevent the aggravation of pressure sore grades when adequate calories are provided through active NST activities, especially for surgery department patients.

Treatment in the ICU also worsened the patient’s pressure sore grade. Patients who did not receive ICU treatment were less likely to have worsening pressure sore grades. This could be because patients who did not receive ICU treatment had low-severity symptoms, and many of them could easily change their body positions, which impacted the pressure sore grade. Since ICU treatment is one of the current indications for NST treatment (6), it is necessary to actively refer ICU–treated patients to the NST and pay particular attention to not aggravating the grade of pressure sores. For reference, the proportion of patients who did not receive ICU treatment was significantly higher in the group with improved pressure sore grades. However, none of the factors significantly affected it when the other variables were considered. Moreover, accurate factor evaluation was not performed because the number of patients was minimal, with only 3.2% of patients having improved pressure sore grades. Since this study was conducted only with patients referred to the NST, it is necessary to examine whether NST activities improve the grade of pressure sores by comparing the pressure sore grades of patients referred to the NST with those not referred to the NST.

The strength of this study is that it was conducted on a relatively large number of patients. However, this study had several limitations. First, it was difficult to generalize the characteristics of the patients because only those who visited a tertiary hospital were included. However, the results of this study are believed to be reliable because it targeted patients who met the NST treatment indications, and these indications were the same in other hospitals. Second, this study could not analyze clinical data such as the patient’s underlying disease or the disease being diagnosed and treated. Therefore, the results should be interpreted with caution because other potential factors may have influenced the results of this study. Finally, this study may have selection bias because the data were collected and analyzed retrospectively.

## 5. Conclusion

Since there is a close relationship between pressure sores and low albumin levels, it is necessary to actively provide overall nutrition, including albumin, by actively implementing NST referrals, especially for patients in the surgery department and those treated in the ICU, as they are at a high risk of pressure sore development and aggravation. Furthermore, since low albumin levels occur more frequently in elderly patients, additional studies are needed to examine whether elderly patients should be included in the NST treatment indications.

## Data availability statement

The original contributions presented in this study are included in the article/Supplementary material, further inquiries can be directed to the corresponding author.

## Ethics statement

This study was approved by the hospital’s Institutional Review Board (2022-09-001). The requirement for consent was waived due to the study’s retrospective nature (2022-09-001). The patients/participants provided their written informed consent to participate in this study.

## Author contributions

JB and HK: conceptualization and writing—review and editing. JB, S-YK, HR, and HK: methodology. HR and JC: software. JC, B-SS, and HK: validation. S-YK: investigation and visualization. JB and HR: resources. B-SS and HK: writing—original draft preparation. HK: supervision and funding acquisition. All authors read and agreed to the published version of the manuscript.
