# Supplementary material for: Characteristics and clinical course of patients referred to the NST
Source: Front Nutr. 2023 Jan 26;10:1071541. doi: 10.3389/fnut.2023.1071541 (PMC9910213; doi:10.3389/fnut.2023.1071541)
Supplement: Supplementary file 1 [file Data_Sheet_1.docx]

Supplementary Material

# Supplementary Tables

# Supplementary Table 1. Differences of patient characteristics according to changes in pressure sore grades

|  | Sore improvement (n=25) | No change (n=697) | Sore aggravation (n=70) | *P value* |
| --- | --- | --- | --- | --- |
| Age (mean) | 74.00±15.11 | 71.70±13.04 | 70.61±14.63 | 0.544 |
| Sex (male) | 13 (52.0) | 424 (60.8) | 46 (65.7) | 0.469 |
| Height | 163.36±10.61 | 163.92±8.79 | 165.35±8.31 | 0.410 |
| Weight | 60.88±9.75 | 59.21±12.72 | 64.61±11.46 | 0.003 |
| BMI | 22.80±2.98 | 21.94±3.88 | 23.59±3.61 | 0.002 |
| Department |  |  |  |  |
| Surgery | 12 (48.0) | 270 (38.7) | 40 (57.1) | 0.009 |
| Internal medicine | 13 (52.0) | 427 (61.3) | 30 (42.9) |  |
| Non-neuro* | 16 (64.0) | 396 (56.8) | 38 (54.3) | 0.702 |
| Neuro* | 9 (36.0) | 301 (43.2) | 32 (45.7) |  |
| Low albumin** | 9 (36.0) | 309 (44.3) | 26 (37.1) | 0.383 |
| EN | 10 (40.0) | 271 (38.9) | 24 (34.3) | 0.744 |
| PN | 13 (52.0) | 399 (57.2) | 40 (57.1) | 0.873 |
| Spontaneous feeding | 3 (12.0) | 87 (12.5) | 7 (10.0) | 0.833 |
| NPO | 13 (52.0) | 356 (51.1) | 38 (54.3) | 0.875 |
| Ascite | 0 (0.0) | 1 (0.1) | 2 (2.9) | 0.002 |
| Edema | 1 (4.0) | 44 (6.3) | 3 (4.3) | 0.722 |
| Jaundice | 0 (0.0) | 2 (0.3) | 0 (0.0) | 0.872 |
| Dialysis | 1 (4.0) | 27 (3.9) | 6 (8.6) | 0.181 |
| Bad appetite | 2 (8.0) | 69 (9.9) | 6 (8.6) | 0.898 |
| Difficulty chewing | 9 (36.0) | 253 (36.3) | 27 (38.6) | 0.930 |
| Difficulty swallowing | 8 (32.0) | 199 (28.6) | 22 (31.4) | 0.829 |
| Diarrhea | 0 (0.0) | 11 (1.6) | 2 (2.9) | 0.584 |
| Constipation | 0 (0.0) | 2 (0.3) | 0 (0.0) | 0.872 |
| ICU treatment | 3 (12.0) | 228 (32.7) | 39 (55.7) | <0.001 |
| APACHE II score | 18.50±0.71 | 14.59±7.91 | 12.70±7.35 | 0.330 |
| Length of ICU stay | 16.33±5.69 | 24.30±20.21 | 31.82±36.83 | 0.142 |
| Average number of weeks to reach the target calories | 1.50±0.85 | 1.60±1.79 | 2.19±2.27 | 0.220 |
| Amounts of improved calories | 29.67±43.05 | 20.36±31.70 | 19.62±35.07 | 0.357 |
| Physicians received NST training | 8 (32.0) | 189 (27.1) | 22 (31.4) | 0.659 |
| Laboratory findings |  |  |  |  |
| WBC (10^3/㎕) | 11.74±5.16 | 11.26±5.62 | 12.00±5.78 | 0.547 |
| Hb (g/dL) | 10.10±2.02 | 10.52±2.10 | 10.15±2.32 | 0.260 |
| Na (mEq/L) | 139.75±5.39 | 139.62±6.64 | 140.87±8.36 | 0.336 |
| K (mEq/L) | 3.58±0.71 | 3.67±0.60 | 3.68±0.62 | 0.750 |
| Cl (mEq/L) | 104.08±4.65 | 104.37±6.93 | 105.23±7.95 | 0.600 |
| Ca (mEq/L) | 8.07±0.53 | 7.97±0.68 | 8.13±0.78 | 0.477 |
| Mg (mEq/L) | 2.10±0.35 | 2.13±0.64 | 2.15±0.30 | 0.942 |
| P (mg/dL) | 3.43±1.22 | 2.94±1.25 | 3.11±2.47 | 0.494 |
| ALT (U/L) | 20.90±15.29 | 40.03±101.39 | 42.48±152.36 | 0.664 |
| AST (U/L) | 33.94±18.65 | 56.71±156.35 | 68.34±247.74 | 0.667 |
| Glucose (mg/dL) | 118.82±53.40 | 136.99±68.15 | 154.93±84.05 | 0.046 |
| Albumin (g/dL) | 3.15±0.57 | 3.13±0.53 | 3.12±0.45 | 0.967 |
| Cholesterol (mg/dL) | 146.69±56.98 | 133.48±46.36 | 120.92±40.82 | 0.134 |
| Triglyceride (mg/dL) | 113.25±65.27 | 111.76±76.84 | 109.58±77.17 | 0.982 |
| CRP (mg/dL) | 7.00±5.36 | 9.53±8.84 | 10.53±8.21 | 0.251 |
| BUN (mg/dL) | 24.33±16.96 | 24.95±17.19 | 25.21±14.84 | 0.976 |
| Cr (mg/dL) | 0.99±1.04 | 1.02±1.09 | 1.09±1.01 | 0.880 |

# Values are the number of patients (%) or mean±standard deviation unless otherwise indicated. *Neuro = Neurology and Neurosurgery, Non-neuro = other departments, **Low albumin = serum albumin ≤3.0 g/dL; BMI= body mass index, EN= enteral nutrition, PN= parenteral nutrition, NPO= nil per os or nothing by mouth, ICU= intensive care unit, APACHE II= acute physiology and chronic health evaluation II, NST= nutrition support team, WBC= White blood cell, Hb=hemoglobin, CRP= C-reactive protein, BUN= Blood urea nitrogen, Cr=creatinine.

# Supplementary Table 2. Differences of patient characteristics according to sex

|  | Male (n=716) | Female (n=455) | *P value* |
| --- | --- | --- | --- |
| Age (mean) | 68.2±14.37 | 71.8±14.19 | <0.001 |
| Height | 169.08±6.25 | 156.00±6.08 | <0.001 |
| Weight | 64.43±12.17 | 54.05±10.68 | <0.001 |
| BMI | 22.48±3.69 | 22.17±3.97 | 0.195 |
| Department |  |  |  |
| Surgery | 353 (49.3) | 197 (43.3) | 0.045 |
| Internal medicine | 363 (50.7) | 258 (56.7) |  |
| Non-neuro* | 395 (55.2) | 230 (50.5) | 0.123 |
| Neuro* | 321 (44.8) | 225 (49.5) |  |
| Low albumin** | 268 (37.4) | 180 (39.6) | 0.465 |
| EN | 247 (34.5) | 179 (39.3) | 0.093 |
| PN | 409 (57.1) | 236 (51.9) | 0.078 |
| Spontaneous feeding | 60 (13.2) | 110 (15.4) | 0.303 |
| NPO | 371 (51.8) | 229 (50.3) | 0.620 |
| Ascite | 5 (0.7) | 1 (0.2) | 0.414 |
| Edema | 24 (3.4) | 32 (7.0) | 0.004 |
| Jaundice | 6 (0.8) | 1 (0.2) | 0.258 |
| Dialysis | 29 (4.1) | 19 (4.2) | 0.916 |
| Bad appetite | 87 (12.2) | 49 (10.8) | 0.472 |
| Difficulty chewing | 232 (32.4) | 167 (36.7) | 0.130 |
| Difficulty swallowing | 176 (24.6) | 141 (31.0) | 0.016 |
| Diarrhea | 15 (2.1) | 6 (1.3) | 0.329 |
| Constipation | 2 (0.3) | 2 (0.4) | 0.647 |
| Pressure sore | 460 (64.2) | 300 (65.9) | 0.555 |
| Pressure sore grade at NST referral |  |  |  |
| Grade 0 | 21 (4.3) | 13(4.2) |  |
| Grade 1 | 327 (67.7) | 207(67.0) |  |
| Grade 2 | 117 (24.2) | 82(26.5) | 0.713 |
| Grade 3 | 13 (2.7) | 6(1.8) |  |
| Grade 4 | 5 (1.0) | 1(0.3) |  |
| Changes in pressure sore grades*** |  |  |  |
| Improved | 13 (2.7) | 12(3.9) |  |
| No change | 424 (87.8) | 273(88.3) | 0.469 |
| Aggravated | 46 (9.5) | 24(7.8) |  |
| ICU treatment | 212 (29.6) | 114(25.1) | 0.090 |
| APACHE II score | 13.69±8.05 | 15.55±7.17 | 0.056 |
| Length of ICU stay | 24.14±21.08 | 24.32±24.62 | 0.947 |
| Average number of weeks to reach the target calories | 1.68±2.07 | 1.55±0.84 | 0.386 |
| Physicians received NST training | 191 (26.7) | 132 (29.0) | 0.384 |
| Laboratory findings |  |  |  |
| WBC (10^3/㎕) | 11.13±5.37 | 11.19±5.32 | 0.851 |
| Hb (g/dL) | 10.85±2.22 | 10.21±2.01 | <0.001 |
| Na (mEq/L) | 140.00±6.90 | 139.78±6.25 | 0.576 |
| K (mEq/L) | 3.75±0.58 | 3.62±0.67 | 0.001 |
| Cl (mEq/L) | 104.80±7.12 | 104.50±6.74 | 0.469 |
| Ca (mEq/L) | 8.06±0.72 | 7.99±0.74 | 0.293 |
| Mg (mEq/L) | 2.12±0.31 | 2.11±0.41 | 0.792 |
| P (mg/dL) | 2.94±1.15 | 3.06±1.54 | 0.371 |
| ALT (U/L) | 39.73±81.23 | 37.42±116.35 | 0.689 |
| AST (U/L) | 60.70±163.03 | 59.14±211.70 | 0.894 |
| Glucose (mg/dL) | 139.31±70.25 | 136.64±66.12 | 0.517 |
| Albumin (g/dL) | 3.20±0.54 | 3.19±0.56 | 0.837 |
| Cholesterol (mg/dL) | 134.00±43.74 | 142.63±46.88 | 0.009 |
| Triglyceride (mg/dL) | 114.33±86.36 | 110.66±74.20 | 0.538 |
| CRP (mg/dL) | 9.36±8.51 | 8.47±8.81 | 0.090 |
| BUN (mg/dL) | 24.97±16.84 | 22.30±15.89 | 0.007 |
| Cr (mg/dL) | 1.12±1.16 | 0.91±1.07 | 0.002 |

# Values are the number of patients (%) or mean±standard deviation unless otherwise indicated. *Neuro = Neurology and Neurosurgery, Non-neuro = other departments, **Low albumin = serum albumin ≤3.0 g/dL, *** Changes in pressure sore grades at the first time of NST referral and discharge; BMI= body mass index, EN= enteral nutrition, PN= parenteral nutrition, NPO= nil per os or nothing by mouth, ICU= intensive care unit, APACHE II= acute physiology and chronic health evaluation II, NST= nutrition support team, WBC= White blood cell, Hb=hemoglobin, CRP= C-reactive protein, BUN= Blood urea nitrogen, Cr=creatinine.

# Supplementary Table 3. Differences of patient characteristics according to ICU treatments

|  | ICU treatment (n=326) | Without ICU treatment (n=845) | *P value* |
| --- | --- | --- | --- |
| Age (mean) | 70.9±13.88 | 69.1±14.57 | 0.060 |
| Sex (male) | 212 (65.0) | 504 (59.6) | 0.090 |
| Height | 163.74±15.72 | 163.14±13.31 | 0.523 |
| Weight | 60.75±13.05 | 59.96±13.35 | 0.347 |
| BMI | 22.35±3.79 | 22.36±3.81 | 0.959 |
| Department |  |  |  |
| Surgery | 152(46.6) | 398 (47.1) | 0.884 |
| Internal medicine | 174 (53.4) | 447 (52.9) |  |
| Non-neuro* | 188 (57.7) | 437 (51.7) | 0.067 |
| Neuro* | 138 (42.3) | 408 (48.3) |  |
| Low albumin** | 148 (45.4) | 300 (35.5) | 0.002 |
| EN | 93 (28.5) | 333 (39.4) | 0.001 |
| PN | 224 (68.7) | 421 (49.8) | <0.001 |
| Spontaneous feeding | 25 (7.7) | 145 (17.2) | <0.001 |
| NPO | 212 (65.0) | 388 (45.9) | <0.001 |
| Ascite | 4 (1.2) | 2 (0.2) | 0.054 |
| Edema | 26 (8.0) | 30 (3.6) | 0.001 |
| Jaundice | 1 (0.3) | 6 (0.7) | 0.681 |
| Dialysis | 22 (6.7) | 26 (3.1) | 0.005 |
| Bad appetite | 21 (6.4) | 115 (13.6) | 0.001 |
| Difficulty chewing | 87 (26.7) | 312 (36.9) | 0.001 |
| Difficulty swallowing | 77 (23.6) | 240 (28.4) | 0.099 |
| Diarrhea | 1 (0.3) | 20 (2.4) | 0.017 |
| Constipation | 1 (0.3) | 3 (0.4) | 1.000 |
| Pressure sore | 238 (73.0) | 522 (61.8) | <0.001 |
| Pressure sore grade at NST referral |  |  |  |
| Grade 0 | 26 (9.6) | 8 (1.5) |  |
| Grade 1 | 169 (62.6) | 365 (69.9) |  |
| Grade 2 | 65 (24.1) | 134 (25.7) | <0.001 |
| Grade 3 | 7 (2.6) | 12 (2.3) |  |
| Grade 4 | 3 (1.1) | 3 (0.6) |  |
| Changes in pressure sore grades*** |  |  |  |
| Improved | 3 (1.1) | 22 (4.2) |  |
| No change | 228 (84.4) | 469 (89.8) | <0.001 |
| Aggravated | 39 (14.4) | 31 (5.9) |  |
| Average number of weeks to reach the target calories | 1.69±1.86 | 1.47±0.93 | 0.204 |
| Number of patients who improved their calories | 250 (76.7) | 612 (72.4) | 0.138 |
| Amounts of improved calories | 23.47±32.95 | 19.33±32.70 | 0.053 |
| Physicians received NST training | 89 (27.3) | 234 (27.7) | 0.893 |
| Laboratory findings |  |  |  |
| WBC (10^3/㎕) | 12.07±6.22 | 10.80±4.93 | 0.001 |
| Hb (g/dL) | 10.33±2.18 | 10.70±2.15 | 0.008 |
| Na (mEq/L) | 139.90±6.96 | 139.92±6.53 | 0.970 |
| K (mEq/L) | 3.69±0.64 | 3.71±0.61 | 0.577 |
| Cl (mEq/L) | 104.17±7.38 | 104.88±6.80 | 0.120 |
| Ca (mEq/L) | 7.97±0.68 | 8.06±0.74 | 0.212 |
| Mg (mEq/L) | 2.12±0.36 | 2.11±0.35 | 0.960 |
| P (mg/dL) | 3.06±1.63 | 2.95±1.13 | 0.453 |
| ALT (U/L) | 46.74±131.45 | 35.78±78.72 | 0.159 |
| AST (U/L) | 73.77±243.02 | 54.81±154.20 | 0.191 |
| Glucose (mg/dL) | 145.74±72.73 | 135.38±66.83 | 0.021 |
| Albumin (g/dL) | 3.10±0.54 | 3.24±0.55 | <0.001 |
| Cholesterol (mg/dL) | 135.79±46.62 | 138.02±44.69 | 0.544 |
| Triglyceride (mg/dL) | 122.16±71.66 | 109.28±84.76 | 0.058 |
| CRP (mg/dL) | 11.24±9.45 | 8.14±8.14 | <0.001 |
| BUN (mg/dL) | 28.41±18.45 | 22.20±15.21 | <0.001 |
| Cr (mg/dL) | 1.26±1.32 | 0.96±1.04 | <0.001 |

# Values are the number of patients (%) or mean±standard deviation unless otherwise indicated. *Neuro = Neurology and Neurosurgery, Non-neuro = other departments, **Low albumin = serum albumin ≤3.0 g/dL, *** Changes in pressure sore grades at the first time of NST referral and discharge; BMI= body mass index, EN= enteral nutrition, PN= parenteral nutrition, NPO= nil per os or nothing by mouth, ICU= intensive care unit, APACHE II= acute physiology and chronic health evaluation II, NST= nutrition support team, WBC= White blood cell, Hb=hemoglobin, CRP= C-reactive protein, BUN= Blood urea nitrogen, Cr=creatinine.
